# High Expressed Emotion (HEE), Assessed Using the Five-Minute Speech Sample (FMSS), as a Predictor of Psychiatric Relapse in Patients with Schizophrenia and Major Depressive Disorder: A Meta-Analysis and Meta-Regression

**DOI:** 10.3390/jcm11216533

**Published:** 2022-11-03

**Authors:** Cristina Mazza, Federico Formica, Stefano Ferracuti, Eleonora Ricci, Marco Colasanti, Silvia Biondi, Alberto Di Domenico, Paolo Roma

**Affiliations:** 1 Department of Neuroscience, Imaging and Clinical Sciences, G. D’Annunzio University of Chieti-Pescara, 66100 Chieti, Italy; 2 Department of Human Neurosciences, Sapienza University of Rome, 00185 Rome, Italy; 3 Department of Psychological, Health and Territorial Sciences, DiSPuTer, G. D’Annunzio University of Chieti-Pescara, 66100 Chieti, Italy

**Keywords:** expressed emotion, Five-Minute Speech Sample, relapse, family, caregiver

## Abstract

Expressed Emotion (EE) describes the tone of a caregiver’s response to a patient with a mental disorder, and it is used to predict relapse. The Five-Minute Speech Sample (FMSS) is a 5-min interview with a caregiver that evaluates only two EE dimensions. The present study aimed at evaluating HEE (High Expressed Emotion) as a predictor of relapse in patients with schizophrenia and major depressive disorder. Six studies were selected for the meta-analysis. In total, the studies included 297 subjects. The analyses included a random effects model; a meta-analysis excluding the study with the smallest sample; a cumulative meta-analysis; a meta-regression with random effects, using patient age and duration of illness as moderators; a leave-one-out meta-analysis; and a funnel plot to estimate publication bias. The FMSS emerged as a valid and reliable tool for measuring EE as a predictor of relapse in patients with schizophrenia and major depressive disorder. Patient age and duration of illness had no significant effect on the results. Future meta-analyses should include more studies to reduce publication bias. EE may be a good predictor of relapse when examined through a fast measurement technique such as the FMSS, which may also be useful to analyze the psychopathological structure of caregivers.

## 1. Introduction

Expressed Emotion (EE) is a construct that describes how a family member or caregiver, when speaking, expresses emotions about another family member with a mental disorder. Numerous definitions of EE have been proposed: Vaughn [1] defined it as “an index of the emotional temperature in the family environment, an indicator of the intensity of the emotional response of the family member, which reveals a lack of affection or his overly intrusive concern”. Bertrando [2] described it as “the measurement of certain characteristics of the family emotional environment in the course of various pathologies, disorders or problems, generally—but not exclusively—psychiatric”. Finally, Peters, Calam, and Harrington [3] proposed it as an “umbrella” comprising a set of behaviors, attitudes, and emotions within familial relationships. In short, EE is the “expressive way of reaction of family members” [4].

The idea that EE might predict mental disorder relapse originated in the 1950s, from the observations of George Brown at the Medical Research Council Social Psychiatry unit in London [5]. Following the introduction of first-generation anti-psychotics (i.e., chlorpromazine), schizophrenic patients began to be discharged from hospitals. Brown noted that patients who were reintegrated into the family context were significantly more likely to present an aggravation of symptoms—sometimes requiring re-hospitalization—relative to patients who were reintegrated into other public structures. This observation led him to hypothesize that patients’ family environment could contribute to predicting a return of their symptoms. EE was thus born as an empirical, predictive index, and the need to measure it led to the development of rating scales and interviews. It was Brown himself who developed the first coding scheme, the Camberwell Family Interview (CFI; [6], later modified by Vaughn & Leff [7,8]), which is still considered the conventional model for measuring EE.

The CFI is administered to the caregiver after the patient’s first hospitalization. The semi-structured interview, which generally takes 30 min to administer, does not include pre-established questions, but merely guidance on eliciting information from the caregiver about the patient’s onset of disease symptoms in the months preceding the worsening of their disease or their re-hospitalization. The interview is audio-recorded and later coded—a process that generally takes 45 min. EE is then estimated on the basis of five coded dimensions: Emotional Overinvolvement, Criticism, and Hostility contribute to raising EE; and Positive Comments and Warmth contribute to reducing EE. Prior to administering the measure, interviewers must complete 1–2 weeks of training.

A second measure of EE, Level of Expressed Emotion (LEE), is a self-report questionnaire comprising 60 items that measure the emotional environment of family relationships. Four parameters are analyzed with reference to the caregiver, with each assessed using 15 items: Intrusiveness, Emotional Response, Emotional Attitude, and Tolerance/Expectations. All items are rated as true or false. Two versions of the LEE are available: one that is administered to caregivers and one that is administered to patients.

The Perceived Criticism (PC) scale is the fastest measure of EE (1 min), but it only assesses a single dimension of EE: Criticism. The measure is administered via two questions—one directed to the caregiver (1), and one directed to the patient (2):

(1) How critical do you think you are to (name of caregiver)?
(2) How critical do you think (name of caregiver) is toward you?

Answers are coded on a 10-point Likert scale ranging from 1 (not at all critical) to 10 (very critical).

The Five-Minute Speech Sample (FMSS) asks the caregiver to talk about their thoughts and feelings about the patient for 5 min, without interruption. Originally developed by Gottschalk and Gleser [9] to assess anxiety, hostility, and hope, it was later proposed by Magana et al. [10] as a specific measure of EE, for which they designed a coding scheme. The FMSS interview is audio-recorded and coded according to Magana’s [11] scoring system. The coding generally takes 20 min to complete. The measure analyzes five parameters of EE: Initial Statement, Relationship, Criticism, Dissatisfaction, and Emotional Overinvolvement (and thereby only assesses the Criticism and Emotional Overinvolvement dimensions). A scale of values is established for each parameter, and scores determine whether respondents exhibit either low (LEE) or high EE (HEE). Training is mandatory to code the FMSS, but not to administer it. The training consists of approximately 20 h of classroom teaching and 15 h of individual study (coding speech samples). Training seminars take place over 5 days and are usually completed within 1–2 weeks.

The present study aimed at analyzing, through a meta-analysis and meta-regression, whether EE, as determined using a fast interview instrument (i.e., the FMSS), could be used to predict relapse in patients suffering from schizophrenic and depressive disorders. To the best of our knowledge, no other meta-analytical research has considered the FMSS alone as a tool for analyzing EE. However, some reviews (e.g., [12]) have analyzed a selection of rating scales.

## 2. Materials and Methods

### 2.1. Search Strategy

**Figure 1:**
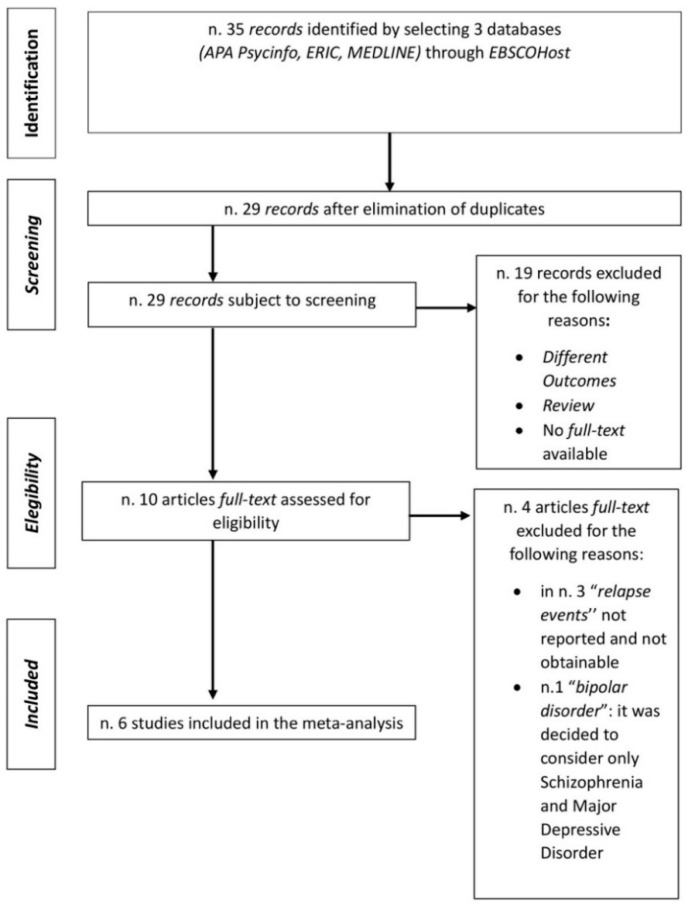
Search strategy flowchart.

Figure 1 presents a flowchart of the main steps followed to select the included articles: identification, screening, eligibility, and inclusion. Initially 35 records were identified within three EBSCOHost databases: APA PsycINFO (1806–2022), ERIC (1966–2022), and MEDLINE (1946–2022). The search was performed using the keywords “Five-Minute Speech Sample” AND “relapse,” considering all publication dates between the inception of the databases and January 2022.

### 2.2. Inclusion and Exclusion Criteria

Following the elimination of duplicates, 29 records were retained and screened for eligibility. A total of 19 (out of 29) [13,14,15,16,17,18,19,20,21,22,23,24,25,26,27,28,29,30,31,32,33,34] were excluded because their outcomes did not relate to the goals of the present meta-analysis, they represented review studies, or their full text was not available. This resulted in 10 articles, of which 4 were excluded because 3 did not report the number of relapse events and 1 dealt with bipolar disorder, which was not considered in the present study due to the limited number of patients.

Thus, a total of six studies were considered in the present meta-analysis and meta-regression.

### 2.3. Sample and Study Characteristics

Table 1 presents the main characteristics considered in the meta-analysis of the selected studies. In particular, the analyses involved 297 subjects with a weighted mean age of 40.2 years (*SD* = 13.37; range = 16.6–70.5). Of these, 221 had a diagnosis of schizophrenia, with 41.1% belonging to HEE families, and 76 had a diagnosis of depression, with 47% belonging to HEE families. As regards the chronicity of disease, three studies reported a chronic condition, one study reported a recent onset, and two studies reported a mixed condition (these studies included patients with both chronic and recent onset conditions.) All the included studies used the FMSS as a measure of EE, and four different measures of relapse:

- Brief Psychiatric Rating Scale (BPRS): the BPRS is used to assess the severity of a range of psychiatric symptoms. It is a 16-item scale, with all items rated on a 7-point Likert scale ranging from 1 (absent) to 7 (extremely severe). Relapse is defined as at least one hospitalization with hallucination, conceptual disorganization, and unusual thought content.
- Global Assessment Scale (GAS): the GAS evaluates overall functioning during a specified time period on a continuum from psychological or psychiatric sickness to health. Relapse is defined as the reappearance or marked exacerbation of schizophrenic symptoms.
- Longitudinal Interval Follow-up Evaluation (LIFE): the LIFE defines relapse as recovery with later major depressive disorder, as determined by research diagnostic criteria (RDC).
- Clinical assessment: relapse is determined by a worsening of psychiatric symptoms and/or an increase in medication dosage.

### 2.4. Statistical Analysis

Typically, the studies included in a meta-analysis differ in their research parameters. This can generate heterogeneous results, rendering the assumption of a common effect size meaningless. Consequently, for the present meta-analysis, we applied a random effects model with a heterogeneity parameter. In contrast to fixed effects models, random effects models assume that effect sizes vary, due to either within-group differences or between-study variance in the study parameters.

We used a restricted maximum likelihood approach to control for bias. To confirm the stability of our parameters, we repeated the analysis, excluding the study with the smallest sample (i.e., [39]), as the random-effects model gave it more weight and it was, therefore, potentially biased. We then performed a cumulative meta-analysis to show how evidence accumulated and conclusions shifted over time. Subsequently, we ran a meta-regression of some random effects to study the association between subject characteristics and effect estimates—and, in particular, the heterogeneity of the effect estimates. Leave-one-out forest plots were used to check the stability of the parameters as individual studies were removed. Finally, we drafted a funnel plot to determine publication bias.

## 3. Results

### 3.1. Meta-Analysis of All Included Studies

**Figure 2:**
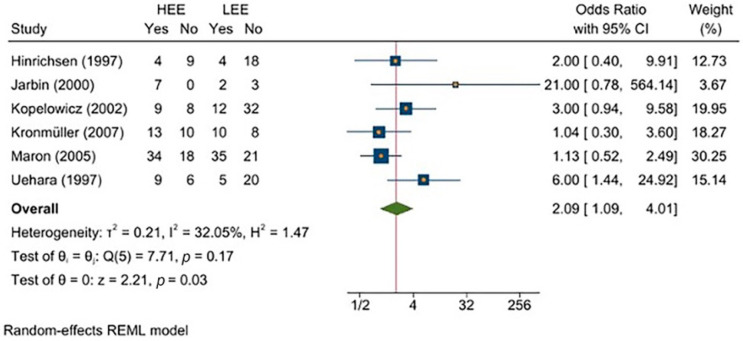
Forest plot of the meta-analysis with all six studies included [35,37,38,39,40,41].

Figure 2 presents a forest plot of the results of the meta-analysis, with all six studies considered. Effect size and confidence interval (CI) values are reported for each study, as is the average or combined effect size and its relative CI. Each study corresponds to a navy square centered on the estimated effect size. The area of each square is proportional to the weight of the respective study (dependent on the number of patients included and the number of events observed) and inversely proportional to the variance of the individual estimate. The horizontal lines connected to the squares represent the CI of the estimated values. The red vertical line is centered on the combined estimate of the effect size, which is represented by the green diamond. The center of the diamond indicates the point estimate and overall effect, and the width represents the confidence interval.

The results showed that HEE may be a strong predictor of relapse, given its overall odds ratio (OR) = 2.09.

The measures of heterogeneity reported in the first line under the plot were:

- *τ*^2^ (tau squared), which indicates variance in the true effect size between studies (i.e., 0.21). This is an absolute measure, as the deviation is measured on the same scale as the effect size index.
- *I*^2^ (I squared), which measures heterogeneity, is expressed as a percentage of the extent to which the observed variance reflects real variability in the effect size. This is a descriptive statistical measure providing information about inconsistency in the study results, rather than a quantitative measure of the dispersion and variability of the true effect size. *I*^2^ = 0 means that the majority of the variability is random; in contrast, *I*^2^ = 32.05 indicates that 32% of the variability in the results of individual studies is explained by real differences between the studies, and not due to chance.
- *H*^2^ (H-squared) is a measure of homogeneity. *H*^2^ = 1 indicates perfect homogeneity, whereas higher values (i.e., *H*^2^ = 1.47) indicate some variability.

The homogeneity test of effect size is presented in the second line of the forest plot. This is calculated on the basis of the Q-stat value, which indicates the ratio between the observed variation and the error associated with the included studies.

The null hypothesis (θ0) holds that the studies share a common effect size: in this case, since the Q-stat value (Q (5) = 7.71) was not statistically significant with *p* > 0.05 (*p* = 0.17), we were forced to accept the null hypothesis and implicitly reject the alternative hypothesis. However, this result was likely due to the limited number of studies considered.

The third line of the forest plot shows whether the effect size is significantly different from 0. In this case, with a value of *z* = 2.21 and a significance level of *p* < 0.05 (*p* = 0.03), the null hypothesis θ0 could be rejected.

### 3.2. Meta-Analysis Excluding the Study with the Smallest Sample

**Figure 3:**
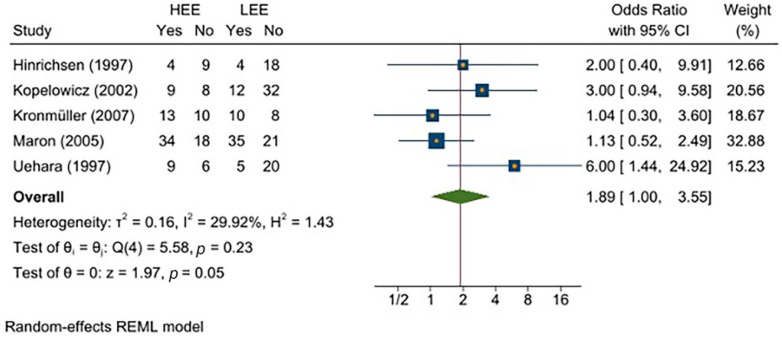
Forest plot of the meta-analysis [35,37,38,39,40,41], excluding the smallest study [39].

To verify the reliability of the parameters, we eliminated the study with the smallest sample [39]. Since the random-effects model gave it more weight, the study was identified as a potential source of bias. The results (presented in Figure 3) were quite encouraging, as the overall effect size was slightly lower (OR = 1.89) than that obtained in the initial analysis (OR = 2.09). The heterogeneity measures also differed slightly from those of the previous analysis (*τ*^2^ = 0.16; *I*^2^ = 29.92%; *H*^2^ = 1.43). Finally, the homogeneity and effect size tests were also statistically significant, although slightly less robust than those obtained in the initial analysis (Q (4) = 5.58, *p* = 0.23; *z* = 1.97, *p* = 0.05).

### 3.3. Cumulative Meta-Analysis

**Figure 4:**
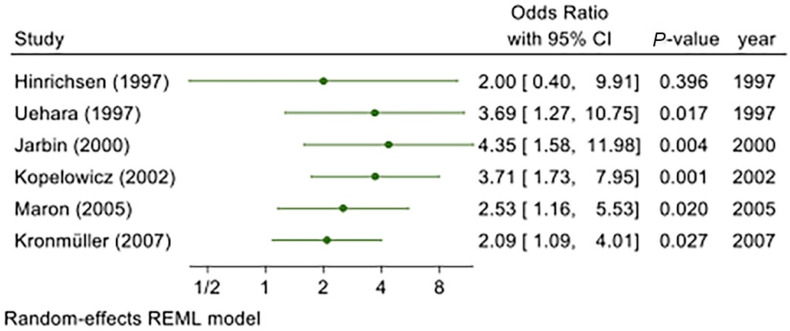
Forest plot of the cumulative meta-analysis [35,37,38,39,40,41].

A cumulative meta-analysis (Figure 4) allowed us to observe how evidence accumulated over time. Initially, the study by Hinrichsen and Pollack [41] showed OR = 2.00 and *p* = 0.369. However, this result was not statistically significant, since it only considered a single study. With the progressive addition of subsequent studies, the results become significant, with *p* < 0.05. Studies published prior to 2003 [35,38,39] showed a high OR (3.69, 4.35, and 3.71, respectively). After this date, OR decreased [[37]; OR = 2.53] until it reached the overall effect size shown in the forest plot in Figure 2 [[40]; OR = 2.09].

### 3.4. Random Effects Meta-Regression

A categorical moderator regression analysis was conducted with the aim of predicting the effect of the duration of illness (chronic vs. non-chronic) and patient age on relapse. The results (Table 2) revealed no statistically significant evidence of a relationship between these variables and relapse, as shown by *p* > 0.05. Therefore, it is unlikely that between-study variability was attributable to these moderators.

### 3.5. Leave-One-Out Meta-Analysis

**Figure 5:**
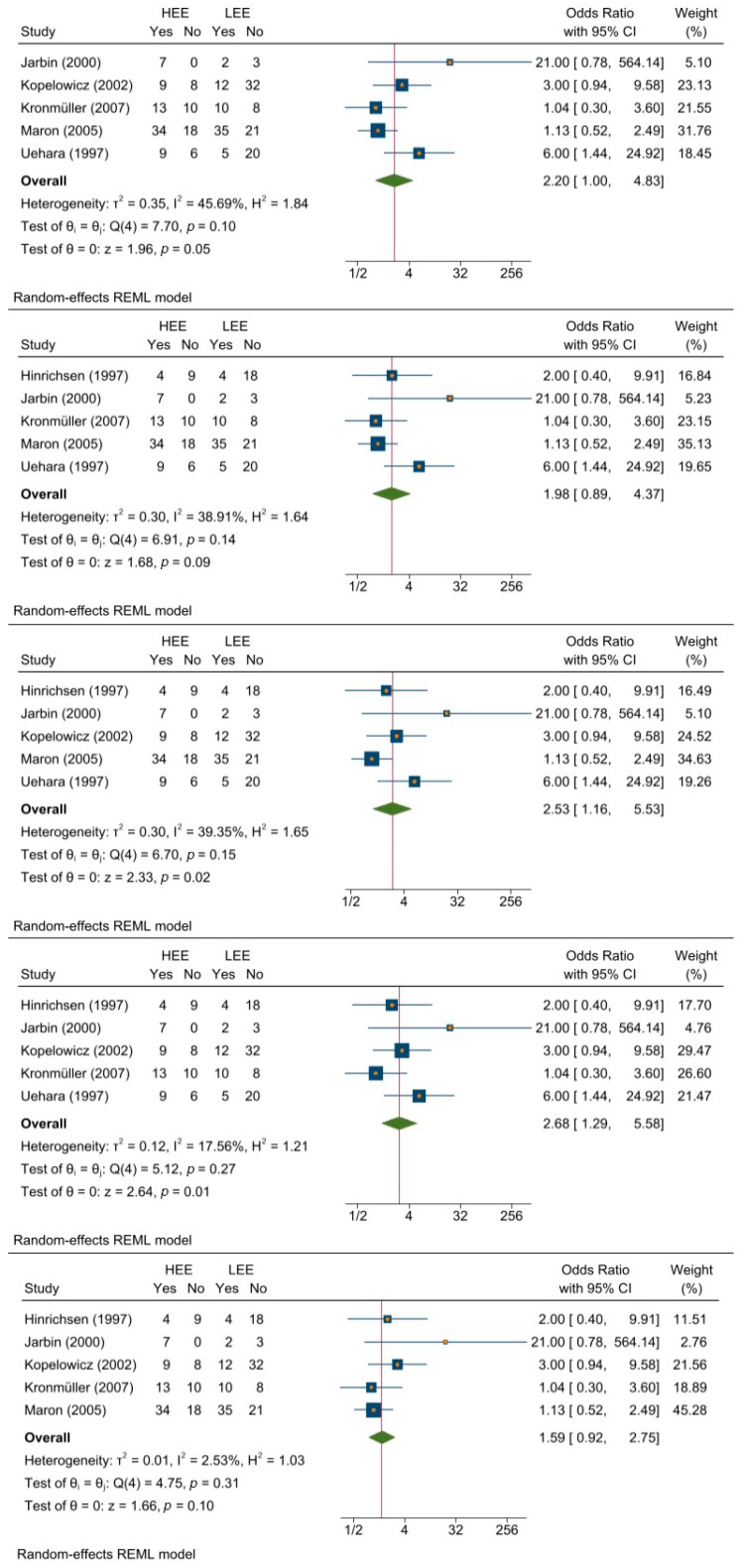
Leave-one-out meta-analysis [35,37,38,39,40,41].

Leave-one-out meta-analysis analyzes each sub-group of studies, with sub-groups defined as the set of all studies, excluding exactly one. This analysis reveals how each individual study affects the overall estimate, and it verifies the reliability and stability of the overall estimate. Figure 5 presents the results of this analysis, excluding, respectively, the studies by Hinrichsen et al. [41], Kopelowicz et al. [38], Kronmüller et al. [40], Marom [37], and Uehara et al. [35]. The subset excluding Jarbin et al. [39] was conducted in the previous analysis (Figure 3), as discussed above.

The smallest effect size was obtained when Uehara et al. [35] was excluded (OR = 1.59); the highest effect size was obtained by excluding Marom [37] (OR = 2.68). Of note, the latter study had the highest number of patients, and therefore, the highest weight; thus, in the meta-analysis comprising all studies, it contributed to attenuating the overall effect size.

### 3.6. Funnel Plot

**Figure 6:**
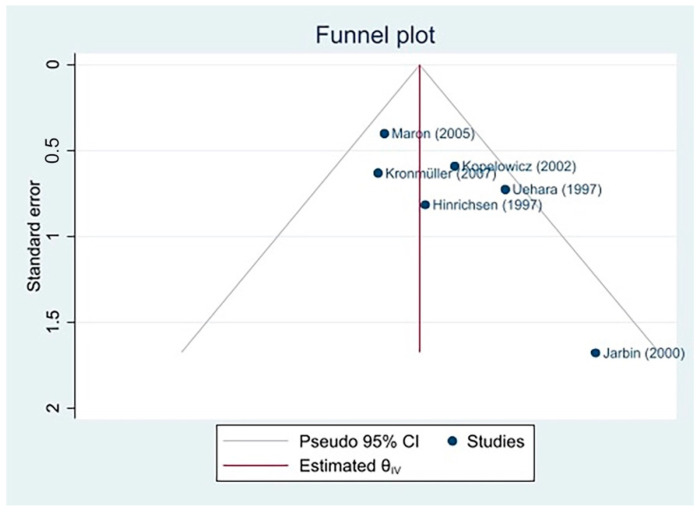
Funnel plot of the meta-analysis conducted with all six studies [35,37,38,39,40,41].

A funnel plot (Figure 6) (also referred to as an inverted funnel plot) was produced to measure publication bias. Publication bias pertains to the failure to publish studies (especially those involving small samples) that fail to find any effect or find insignificant effects. In a funnel plot, if effect size estimates are symmetrically distributed around the average effect size, publication bias can be excluded. Typically, less variation is displayed by larger studies.

In Figure 6, the *x*-axis displays the effect size of each study included in the meta-analysis, while the *y*-axis presents the standard error, with inverted values. The red vertical line represents the true effect size. A symmetrical distribution around the red line was not appreciable, as the number of studies was very low.

As displayed in the forest plot of the leave-one-out meta-analysis excluding Jarbin et al. [39], this study was a strong source of bias for the effect size estimate—also given its standard error, which was almost at the limit of the range considered.

## 4. Discussion

The findings of the present meta-analysis suggest that the FMSS is a valid and reliable tool for evaluating EE as a predictor of relapse in patients with schizophrenia and major depressive disorder. The results are encouraging: the average effect size of EE on relapse was statistically significant, with OR = 2.09; this suggests that EE may be a good predictor of relapse when examined through a fast measurement technique such as the FMSS.

The validity of the results of the first analysis was verified by excluding the study with the smallest sample size, which distorted the overall effect size. The resulting OR remained significant and did not suffer a net decrease. The results also showed that moderators such as patient age and duration of the pathological condition (i.e., chronic vs. non-chronic) were not significantly related to relapse. However, contrary to these findings, the literature reports evidence of the impact of EE on relapse in schizophrenic patients [12,42] related to disease duration. The different findings that emerged in the present meta-analysis likely resulted from the small number of studies considered.

Several studies have shown that the FMSS is a more convenient measure of EE than the CFI. Training in coding the FMSS to measure EE takes approximately 20 h, as opposed to 70 h for the CFI. Furthermore, the actual practice of FMSS coding takes approximately 20 min, whereas CFI coding requires 4–5 h [43]. Concerning administration time, the FMSS takes 5 min, and the CFI involves approximately 2 h. However, FMSS scores for EE correspond to those produced by the CFI, although caregivers classified as HEE by the CFI tend to be under-identified by the FMSS, thus, producing false negatives [44].

The rapid administration of the interview and coding, and thus, the easy accessibility (without the sacrifice of the interview method) of the FMSS make it a convenient measure for EE. Compared to the CFI, which represents the conventional instrument, the FMSS assesses the dimensions of Criticism and Emotional Overinvolvement, only, and the categories of Initial Statement, Relationship, and Dissatisfaction. It does not consider the dimensions of Warmth and Positive Remarks, which are considered irrelevant for the assessment of EE, as well as Hostility, given that the brevity of the interview results in a relative infrequency of hostile comments [45].

Moreover, in light of the FMSS interview structure, based on a free association of ideas, the measure may not only assess EE, but it may also analyze the psychopathological structure of caregivers themselves. As a consequence, it may be possible to apply family psychoeducation interventions, jointly with patient’s pharmacological and psychological treatments, to manage emotional reactions, both to minimize relapse in patients and to benefit caregivers. Such interventions might reinforce dimensions of EE that are configured as protective factors and reduce domains described as risk factors, with the ultimate goal of effecting a renewed therapeutic relationship in the family context.

Psychoeducation consists of the provision of information (about the disease, including its possible course and the consequences of non-adherence to pharmacological therapy) and instructions to prevent and manage mental and psychological disorders. This is particularly relevant, as poor compliance with pharmacological therapy has been identified as the primary cause of relapse [46]. Over the past 10 years, several studies have been conducted with the aim of reducing detrimental EE domains (i.e., Emotional Overinvolvement, Criticism, Hostility) through family psychoeducation, and the effect on global EE has been equivocal. However, a very recent meta-analysis [12] showed that, with schizophrenic patients, reduction in Criticism was associated with a reduction in global EE and a lower risk of relapse. Furthermore, psycho-educated families could benefit from cognitive-behavioral family psychotherapy, as illustrated by two recent reviews showing that this form of therapy is effective in promoting and improving family dynamics.

Finally, it would be helpful to evaluate EE as a predictor of not only relapse, but also psychosis onset.

## 5. Conclusions

Despite the encouraging results obtained in the present meta-analysis, the findings should be read in consideration of the small number of studies included, which represents a significant limitation. The small number of studies resulting from the identification, screening, and eligibility stages did not allow us to make inferences about the influence of Western/Eastern culture. However, it is important to underline that the literature suggests how Western culture encourages more direct expression and how individuals tend to have more emotional arousal [12,47]. Of considerable interest in this direction is the study by Bertrando et al. [48], which shows that different cultures are associated with different levels of EE. It is evident how moving toward traditional cultures and rural environments decreases the rate of EE. Additionally, as displayed in the funnel plot (Figure 6), a symmetrical distribution of effect sizes around the true effect size was not evident. This suggests the presence of significant publication bias, which could be addressed in future research via the inclusion of a larger number of studies. Another limitation of the research is that the included studies employed a variety of definitions of relapse.

Concerning the FMSS, an important limitation pertains to the mandatory training required for coding, which prevents its immediate application by clinicians. Moreover, the interview method, which implies active collaboration with caregivers, may show poor compliance in the context of the psychiatric relapse of a family member.

## Figures and Tables

**Table 1:** Characteristics of the six studies examining the association between EE and relapse in patients with schizophrenia and major depression.

Study	Geographical Area	Number of Subjects	Age	Disease	Disease Chronicity	EE Measure	Relapse Measure	Follow-Up	Relapse Events (Relapsed Subjects/Total Subjects)
Uehara et al. [35]	Japan	40	28.5	Schizophrenia	Chronic	FMSS	GAS	9 months	HEE: 9/15
									LEE: 5/25
Marom et al. [36]	Israel	108	35.4	Schizophrenia and related disorders	Chronic	FMSS	BPRS	9 months	HEE: 23/52
									LEE: 13/56
Marom [37]	7-year follow-up study of Marom et al. [36]							7 years	HEE: 34/52
									LEE: 35/56
Kopelowicz et al. [38]	American study on Caucasians and Mexican-Americans	61 (17 Caucasians, 44 Mexican-Americans)	35.7	Schizophrenia and related disorders	Chronic	FMSS	Clinical assessment	2 years	HEE: 9/17
									LEE: 12/44
Jarbin, Gråwe & Hansson [39]	Sweden	12	16.6	Schizophrenia and related disorders	Recent onset	FMSS	Clinical assessment	2 years	HEE: 7/7
									LEE: 2/5
Kronmüller et al. [40]	Germany	41	44.7	Depression	Mixed condition	FMSS	LIFE	10 years	HEE: 13/23
									LEE: 10/18
Hinrichsen & Pollack [41]	USA	35	70.5	Depression	Mixed condition	FMSS	LIFE	1 year	HEE: 4/13
									LEE: 4/22

Abbreviations: GAS (Global Assessment Scale); BPRS (Brief Psychiatric Rating Scale); LIFE (Longitudinal Interval Follow-up Evaluation); FMSS (Five-Minute Speech Sample); HEE (High Expressed Emotion); LEE (Low Expressed Emotion).

**Table 2:** Meta-regression results on categorical moderators.

Moderator	Number of Studies	OR (Std. Err)	*p*-Value
Duration of condition			0.790
(Chronic)	3	1.23	
(Non-chronic)	3	(2.177)	
Age	6	0.98	0.29
		(0.96)	

## Data Availability

The datasets used and/or analyzed during the current study are available from the corresponding author on reasonable request.
